# Selective Vapor‐Phase Formic Acid Decomposition Over Carbon‐Supported Rhenium Catalysts with Metallic, Carbide, and Oxide Rhenium Phases

**DOI:** 10.1002/open.202500412

**Published:** 2025-09-22

**Authors:** Claudio Contreras‐Díaz, Verónica Naharro‐Ovejero, Claudio Araya‐López, Juan Seguel, Marcos Flores, Vicente Diaz, Néstor Escalona, Ana Belén Dongil

**Affiliations:** ^1^ Departamento de Ingeniería Química y Bioprocesos Pontificia Universidad Católica de Chile Avenida Vicuña Mackenna 4860 Macul Santiago Chile; ^2^ Millennium Nuclei on Catalytic Processes Towards Sustainable Chemistry (CSC) Santiago Chile; ^3^ Instituto de Catálisis y Petroleoquímica CSIC Cantoblanco 28049 Madrid Spain; ^4^ Departamento de Física Facultad de Ciencias Físicas y Matemáticas Universidad de Chile Avenida Blanco Encalada 2008 Santiago Chile; ^5^ Departamento de Química Física Facultad de Química y de Farmacia Pontificia Universidad Católica de Chile Santiago 7820436 Chile

**Keywords:** carbon supports, decomposition, formic acid, heterogeneous catalysis, Re phases, rhenium

## Abstract

Formic acid is obtained as a byproduct of biomass pyrolysis and is used as a liquid organic hydrogen carrier due to its low decomposition temperature, enabling hydrogen production under mild conditions with noble metals. The decomposition of FA in the vapor phase using different rhenium phases (metal, carbide, and oxide) supported on graphite and carbon nanotubes was studied within a temperature range of 80–220 °C, in a fixed‐bed reactor with a space velocity of 651 mL g_cat_ h^−1^. The catalysts were characterized by N_2_ adsorption–desorption, H_2_‐temperature‐programmed reduction, transmission electron microscopy, temperature programmed desorption‐ammonia, temperature programmed reaction‐methanol, X‐ray diffraction, and X‐ray photoelectron spectroscopy. Graphite‐supported catalysts achieved higher activity than carbon nanotube‐supported ones, due to the higher rhenium dispersion on graphite. Catalytic reactions revealed that ReC/G exhibited superior performance at lower temperatures per active site, attributed to the rhenium carbide phase. High selectivity toward CO_2_ was observed across all catalysts, except for ReOx/G at lower temperatures, where differences in active site characteristics likely influenced performance. ReC/G displayed the highest intrinsic activity, highlighting rhenium carbide as a more active phase than metallic or oxide rhenium.

## Graphical abstract

Carbon‐supported rhenium catalysts with metallic, carbide, and oxide phases were evaluated for vapor‐phase formic acid decomposition. Rhenium carbide exhibited the highest intrinsic activity and superior CO_2_ selectivity, while graphite provided enhanced dispersion compared to carbon nanotubes, highlighting phase‐dependent performance in hydrogen carrier applications.

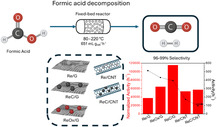

## 1 Introduction

Hydrogen is a critical energy vector due to its high energy density.^[^ ^1^ ^]^ Environmental cleanliness and natural abundance on Earth.^[^ ^2^ ^]^ It is widely utilized in diverse industrial applications, including the hydrogenation of platform molecules,^[^ ^3^ ^]^ fuel cells,^[^ ^4^ ^]^ and ammonia production.^[^ ^5^ ^]^ One promising method of hydrogen storage involves molecules known as liquid organic hydrogen carriers (LOHCs), which can reversibly store and release hydrogen under mild conditions.

Among LOHCs, formic acid (FA) has recently attracted significant attention due to its ability to release hydrogen at low temperatures. However, current production of FA is primarily based on fossil feedstocks.^[^ ^6^ ^]^ As an alternative, FA is also generated as a valuable byproduct in various biorefinery processes and exhibits several advantageous properties, such as nontoxicity, biodegradability, and favorable energy density, making it a strong candidate for hydrogen storage.^[^ ^7^ ^]^ The thermal decomposition of formic acid can proceed via two main pathways: (HCOOH → H_2_ + CO_2_) or dehydration (HCOOH → CO + H_2_O). The preferred pathway, dehydrogenation, produces hydrogen and carbon dioxide. In contrast, the production of carbon monoxide, which is a well‐known poison for catalysts, particularly those commonly used in fuel cells, such as Pd and Pt. The pathway selected depends on factors such as the catalyst, formic acid concentration, and reaction temperature.

Selective H_2_ production from formic acid has been studied over different catalysts, mostly on noble metals such as Pd,^[^ ^8^ ^]^ Pt,^[^ ^9^ ^]^ Au,^[^ ^10^ ^]^ and Ru,^[^ ^11^ ^]^ among others. Nevertheless, the high cost and scarcity of noble metals limit their practical application. As a result, research has long focused on identifying alternative catalysts, such as non‐noble metals or nonmetals, to replace the expensive noble metals traditionally used in the dehydrogenation of formic acid (HCOOH). In this context, transition metal oxides, including materials like Al_2_O_3_,^[^ ^12^ ^]^ Fe_2_O_3_,^[^ ^13^ ^]^ and ZrO_2_,^[^ ^14^ ^]^ have been explored as viable catalysts for this reaction. In this context, transition metal carbides like Mo_2_C exhibit noble metal‐like catalytic behavior due to their unique electronic structure, where carbon insertion modifies the d‐band properties, enhancing activity in hydrogenation reactions while maintaining stability, mirroring precious metals but at reduced cost.^[^ ^15^ ^]^ Overall, Mo_2_C^[^ ^16^, ^17^ ^–^ ^18^ ^]^ and Mo_2_N^[^ ^19^ ^,^ ^20^ ^]^ Have been extensively used in the dehydrogenation of formic acid with the selectivity of H_2_ over 80%. However, Mo_2_C and Mo_2_N have a low surface area, which implies a lower conversion of the reaction.^[^ ^21^ ^]^ In this respect, the use of carbon as support is attractive for molybdenum carbide catalysts due to its higher surface area, low cost, and its ubiquitous nature. Graphite‐supported Mo_2_C exhibits improved catalytic activity compared to bulk Mo_2_C, attributed to the increased dispersion of the active phase.^[^ ^22^ ^]^

Rhenium has been extensively used in the hydrogenation of different biomass derivatives such as 4‐(furyl)‐3‐buten‐2‐one,^[^ ^23^ ^]^ 2‐methoxyphenol,^[^ ^24^ ^]^ glycerol,^[^ ^25^ ^]^ levulinic acid,^[^ ^26^ ^]^ and CO_2_ methanation.^[^ ^27^ ^]^ In this concern, different rhenium phases, such as carbide,^[^ ^28^ ^]^ sulfide,^[^ ^29^ ^]^ and oxide,^[^ ^30^ ^]^ has been used to hydrogenate biomass derivatives. These results and DFT modeling^[^ ^31^ ^]^ suggest that rhenium is an attractive option to replace molybdenum carbide in the decomposition of formic acid with a higher conversion. However, to date, there have been no studies of Re in the dehydrogenation of formic acid. Furthermore, carbon supports such as high‐surface‐area graphite and carbon nanotubes could be interesting options for studying the effects of support morphology.

In this study, different rhenium phases (metallic, oxide, and carbide) supported on carbon nanotubes and high‐surface‐area graphite for the dehydrogenation of formic acid in a vapor‐phase fixed‐bed reactor system were studied.

## 2 Results and Discussion

**Figure 1:**
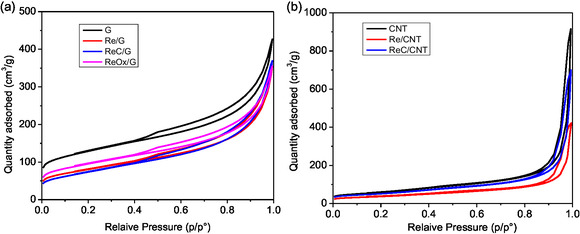
N_2_ adsorption/desorption of rhenium‐based catalysts supported on a) graphite and b) carbon nanotubes.

The N_2_ adsorption/desorption isotherms of the rhenium‐based catalysts are presented in **Figure** **1**. Figure 1a shows the N_2_ isotherms for the rhenium‐based catalysts supported on graphite. All these catalysts displayed a type IV isotherm with an H4 hysteresis loop, according to IUPAC criteria,^[^ ^32^ ^,^ ^33^ ^]^ which is characteristic of mesoporous materials with slit‐shaped pores. Conversely, Figure 1b displays the N_2_ isotherms for the rhenium‐based catalysts supported on carbon nanotubes. These catalysts exhibited a type IV isotherm with an H1 hysteresis loop, indicative of mesoporous materials with cylindrical pores, as typically observed in carbon nanotubes.^[^ ^34^ ^]^ **Table** **1** summarizes the textural properties derived from the N_2_ isotherms, including the specific surface area, pore volume, and average pore diameter of the samples.

**Table 1:** Textural properties of supports and rhenium‐based supported catalysts.

Catalysts	wt% Re loading	*S*_BET_ [m^2^ g^−1^]	V_p_ [cm^3^ g^−1^]	D_p_ [nm]
CNT		212	1.39	29.2
ReC/CNT	10	191	1.43	30.0
Re/CNT	10	127	0.82	25.9
G		430	0.87	5.9
Re/G	10	283	0.51	7.3
ReC/G	10	266	0.56	8.4
ReOx/G	10	331	0.54	6.5

It is observed that a diminution of surface area is in agreement with pore volume diminution and pore diameter change compared with the pristine support. This behavior suggests partial pore blocking due to the Re deposition on the surface. It is interesting to note that the surface area diminished with the higher temperature treatments such as observed in all catalysts, except for ReOx/G. Figure S1, Supporting Information further supports this observation, as the pore size distribution confirms pore filling, evidenced by a decrease in dV/dlogw in all catalysts.

### 2.1 XRD

**Figure 2:**
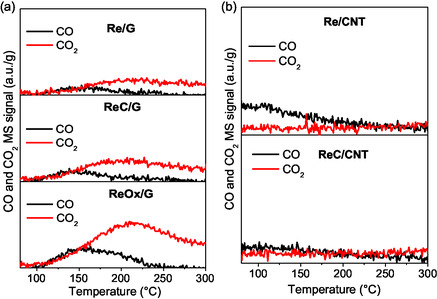
28 and 48 *m*/*z* MS signal of CO and CO_2_, respectively, from TPRe‐MeOH of Re‐based catalysts supported on a) graphite and b) carbon nanotubes.

**Figure 3:**
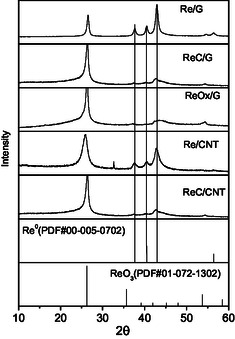
X‐ray patterns of rhenium‐supported catalysts.

The X‐ray diffraction (XRD) analysis for each catalyst is depicted in **Figure** **2**. All catalysts exhibit characteristic diffraction peaks of graphite at ≈2*θ* = 26°, 43°, and 55°, corresponding to the (002), (100)/(101), and (004) planes, respectively. On the ReOx/G catalyst, diffraction peaks associated with rhenium oxide (ICCS, PDF no. 01 072–1302) are barely visible, suggesting the formation of particles below the detection limit of the equipment (≤4 nm). In the case of Re/G and Re/CNT catalysts, distinctive diffraction peaks at 37.6°, 40.5°, and 42.9° were observed, which are attributed to the hexagonal close‐packed (HCP) phase of metallic rhenium (ICCS, PDF no. 00 005–0702). In contrast, for ReC/G and ReC/CNT catalysts, a shift in the diffraction patterns to lower values was compared to the metallic rhenium catalysts (it can be observed well in Figure S2, Supporting Information). This shift is indicative of carbon incorporation into the metallic Re matrix, suggesting the formation of rhenium carbide, as previously reported by Blanco et al.^[^ ^28^ ^]^ (**Figure** **3**).

### 2.2 Temperature‐Programmed Reduction

**Figure 4:**
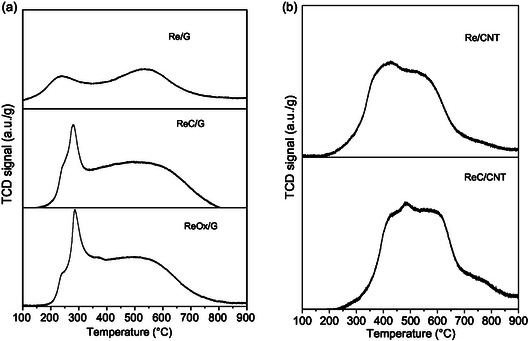
Temperature‐programmed reduction of rhenium‐based catalysts supported on a) graphite and b) carbon nanotubes.

H_2_‐TPR profiles of rhenium‐based catalysts are depicted in **Figure** **4**. Figure 4a presents the profile of rhenium catalysts supported on high surface area graphite, which displays a reduction peak at 300 °C with a shoulder at 250 °C and a broader signal centered at 550 °C. However, Re/G catalyst presents a low intensity reduction peak at 230 °C, suggesting incomplete reduction to metallic rhenium. The first reduction peak was attributed to the reduction of rhenium oxide and carbide species.^[^ ^35^ ^]^ The second peak was associated with the reduction of support, in agreement with the CH_4_‐MS signals included in Figure S3, Supporting Information.

Figure 4b depicts the profiles of rhenium‐based catalysts supported on carbon nanotubes, which show a broad reduction peak with a maximum at 450 °C with a shoulder at 550 °C. These two reduction peaks are assigned to the reduction of rhenium oxide species and the reduction of the support, respectively, similar to the graphite‐supported samples. Notably, in Figure 4b, the first reduction peak of rhenium‐based catalysts supported on carbon nanotubes shifts to higher temperatures compared to the graphite‐supported catalysts.^[^ ^36^ ^]^ This could be related to stronger interactions between Re and the CNT functional groups.

The mass signal corresponding to CH_4_ (*m*/*z* = 15) is shown in Figure S3, Supporting Information, for rhenium‐based catalysts supported on a) graphite and b) carbon nanotubes. CH_4_ formation is attributed to the gasification of the support, catalyzed by metal or metal carbide species. Based on the approach developed by Oyama et al.,^[^ ^37^ ^]^ four distinct temperature zones can be identified: adsorptive carbon (200–300 °C), carbide (460–490 °C), reactive pyrolytic carbon (600–690 °C), and graphitic carbon (700–800 °C). In Figure S3a, Supporting Information, all catalysts exhibit a broad feature centered at 550 °C, primarily attributed to reactive pyrolytic carbon and graphitic carbon. Additionally, the ReC/G catalyst displays a shoulder at 430 °C, which is likely associated with the carbide species, in agreement with XRD results. In Figure S3b, Supporting Information, a strong peak centered at 750 °C is observed for both catalysts, corresponding to graphitic carbon. Notably, the ReC/CNT catalyst also exhibits a feature centered around 420 °C, attributed to carbide species.

### 2.3 Transmission Electron Microscopy

**Figure 5:**
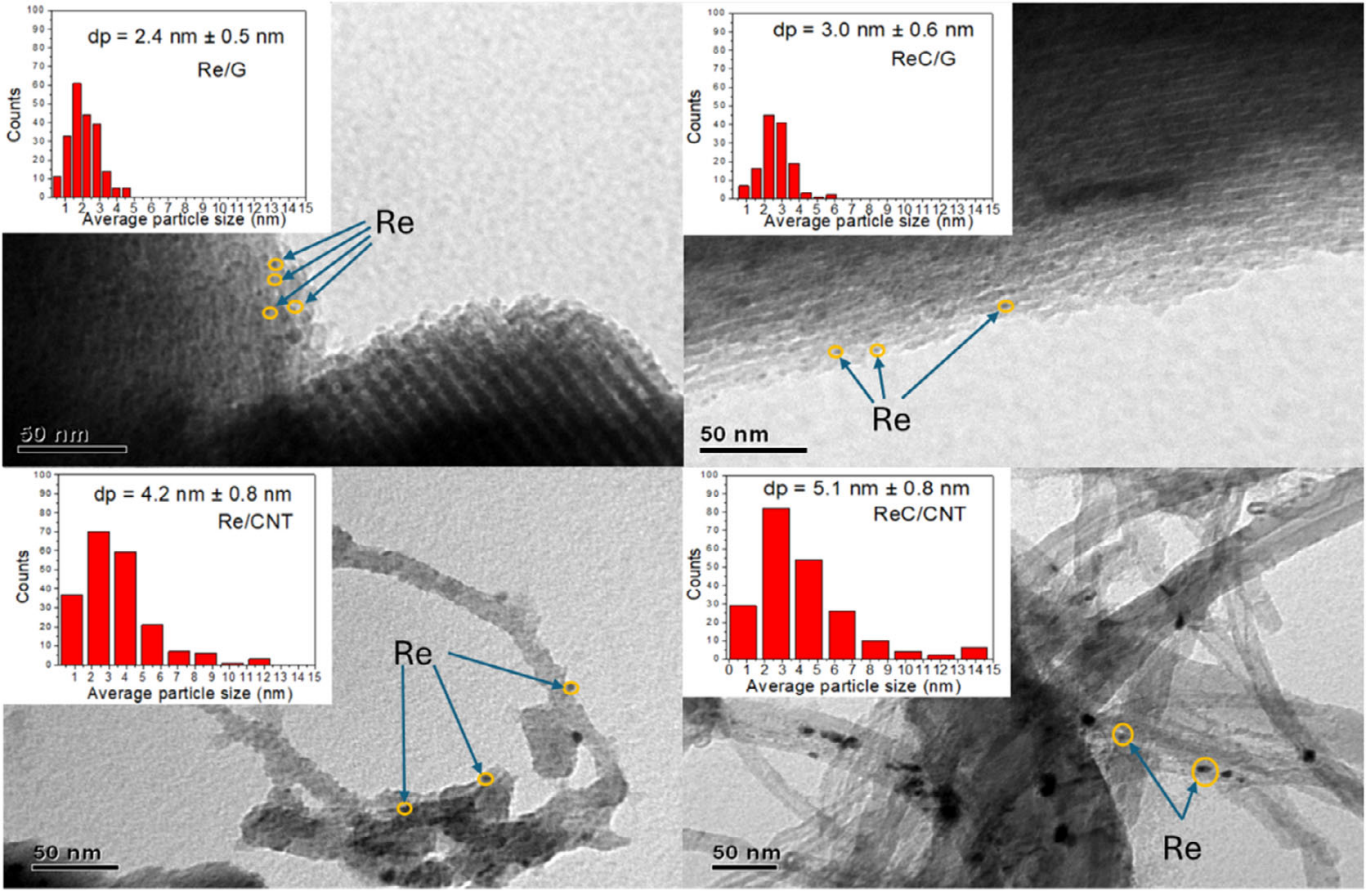
TEM images and particle size distribution of the rhenium‐based catalysts.

**Figure** **5** shows micrographs of the rhenium‐based catalysts supported on carbon materials, along with the corresponding histograms of the Re particle size distribution on the carbon supports. Re/G and ReC/G catalysts exhibit hemispherical particles, attributed to metallic Re and carbidic Re phases, respectively. Re/CNT and ReC/CNT catalysts exhibit larger hemispherical particles with different particle sizes. It is worth noting that although only representative images are shown, the particle size distributions were obtained from at least 10 micrographs per sample. In the case of Re/CNT and ReC/CNT, the curvature and contrast of the carbon nanotube support limit the visibility of Re particles in a single image, which may explain the lower visual clarity compared to the graphene‐based samples. The mean Re particle size distribution, ranked from smallest to largest, follows the trend: Re/G < ReC/G < Re/CNT < ReC/CNT.

Unfortunately, TEM could not be used to estimate the particle size of ReOx/G catalyst because the particles are not distinguishable from the support, as shown in Figure S4, Supporting Information. This limitation is likely due to the insufficient electron density contrast between the rhenium oxide species and the graphite support, which makes it difficult to distinguish the oxide phase. Additionally, Re_2_O_7_ species tend to form a thin monolayer of Re, further complicating its detection, a behavior consistent with reports by Oikawa et al.^[^ ^38^ ^]^ for Re_2_O_7_(7%)/Al_2_O_3_. Similar findings were documented by Yide et al.,^[^ ^39^ ^]^ where rhenium oxide on Al_2_O_3_ formed a monolayer up to ≈0.35 nm^2^ per Re atom at 18 wt% Re_2_O_7_ loading.

### 2.4 TPRe‐MeOH

**Figure 6:**
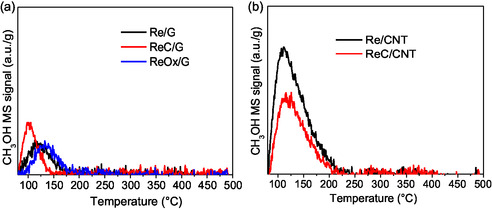
31 *m*/*z* MS signal of CH_3_OH from TPRe‐MeOH of Re‐based catalysts supported on a) G and b) CNT.

**Figure** **6**, S5, and S6, Supporting Information, show the results of TPRe‐MeOH analysis for rhenium catalysts supported on carbon materials. The mass signal corresponding to MeOH (*m*/*z* = 31) is shown in Figure 6a for catalysts supported in graphite (G) and in Figure 6b for those supported on carbon nanotubes (CNT).

Figure 6a shows the desorption peak associated with the non‐reacted MeOH; all graphite‐supported catalysts exhibit a single peak centered between 100 and 125 °C, indicating similar site strength. The higher signal at the intensity of this peak in the ReC/G catalyst peak suggests a lower number of active sites compared to Re/G and ReOx/G. In Figure 6b, CNT‐supported catalysts show a similar single peak at ≈125 °C but with greater intensity than their graphite‐supported counterparts. This suggests that graphite‐supported catalysts possess fewer active sites or lower activity overall compared to CNT‐supported catalysts.

Figure S6, Supporting Information, focuses on the redox sites of rhenium‐based catalysts. Both Figure S6a,b, Supporting Information, displayed a single peak centered between 100 and 150 °C, with the ReOx/G catalyst exhibiting the most intense peak. This indicates its superior ability to oxidize CH_3_OH to HCOH.^[^ ^40^ ^]^ The redox sites strength appears similar across all catalysts, as no significant variation in the formation temperature of HCOH is observed. Regarding acid sites, Figure S5, Supporting Information, shows no formation of dimethyl ether, suggesting a low presence of these sites in all the tested catalysts. This is supported in previous works by Blanco, et al., where weak acid sites were observed by TPD‐NH_3_ over Re catalysts supported with a centered peak at 200 °C.^[^ ^36^ ^]^ Furthermore, it is known that the dehydration of MeOH to DME occurs in Bronsted acid sites,^[^ ^41^ ^]^ suggesting that rhenium‐based catalysts have Lewis acid sites. On the other hand, Figure S7, Supporting Information, shows that the TPD‐NH_3_ of Re/G and ReOx/G catalysts were for both catalysts; a well‐defined NH_3_ desorption peak at about 200 °C is observed and assigned to weak acid sites, characteristics of rhenium species. The quantity of total acid sites is summarized in Table S1, Supporting Information, for Re/G and ReO_*x*_/G catalysts. ReOx/G possesses approximately twice the number of acid sites compared to Re/G. This could be attributed to Lewis acid sites created in the process of formation of vacancies, suggested by XPS, as in previous works in which a rhenium oxide peak at 45.4 eV is associated with a Re_2_O_7_ species with oxygen vacancies forming a mixed oxide between Re_2_O_7_ and ReO_3_.^[^ ^23^ ^]^

Figure 2 presents the mass signals of carbon monoxide (*m*/*z* = 28) and carbon dioxide (*m*/*z* = 48), which are associated with basic sites.^[^ ^42^ ^]^ In Figure 2a, for graphite‐supported catalysts, the temperature at which CO is observed is 150 °C, and the temperature at which CO_2_ is observed is 200 °C for all the G catalysts, indicating similar basic site strength. In contrast, Figure 2b shows no CO or CO_2_ formation for rhenium catalysts supported on CNTs, suggesting that their basic sites are insufficient to transform MeOH into CO or CO_2_. The increased intensity of the ReOx/G signal indicates a larger amount of basic sites compared to Re/G and ReC/G catalysts. It cannot be ruled out that the carbonous supports could contribute an amount of CO and CO_2_ from partial decomposition of the surface functional groups, as it was observed in the TPD‐He in Figure S8, Supporting Information.

### 2.5 XPS

**Figure 7:**
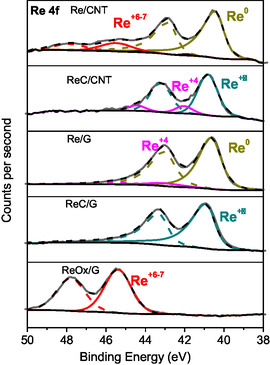
Re 4f core levels spectra of the carbon‐supported rhenium catalysts.

**Figure** **7** presents the XPS deconvolutions in the Re 4f region for the rhenium‐based catalysts. The figure shows that Re/CNT, ReC/CNT, and ReC/G catalysts displayed two partially overlapped doublets. Meanwhile, Re/G and ReOx/G catalysts present only one doublet peak, each one containing Re 4f_7/2_ and Re 4f_5/2_ peaks. **Table** **2** summarizes the binding energies (BE) of the Re 4f_7/2_ core level, their respective percentages, and the atomic surface ratio of Re/C and Re/O for rhenium‐based catalysts supported on carbon materials.

**Table 2:** Binding energies (BE, eV) of the Re 4f_7/2_ components detected on the catalysts.

	Species	ReC/CNT	Re/CNT	ReC/G	Re/G	ReOx/G
BE, eV [%] Re 4f_7/2_	**Re** ^**0**^	–	40.4(91)	–	40.6(94)	
	**Re** ^***δ*+**^	40.8(86)	–	40.9(100)	–	–
	**Re** ^**2+**^	–	–	–	–	–
	**Re** ^**4+**^	42.0(14)	–	–	42.5(6)	–
	**Re** ^**6–7+**^	–	45.6(8)	–	–	45.4(100)
Re/C		0.0017	0.0032	0.0034	0.0138	0.0067

In the table, it is observed that Re/CNT and Re/G catalysts displayed a peak at 40.4 and 40.6 eV that could be attributed to metallic rhenium.^[^ ^43^ ^]^ Re/CNT and ReOx/G catalysts present a peak at 45.4 and 45.6 eV that could be assigned to Re^+7^ with oxygen vacancies, as suggested by Leiva et al.^[^ ^29^ ^]^ ReC/CNT and ReC/G catalysts show a peak at 40.8 and 40.9 eV, which can be attributed to Re^*δ*+^ in RexC species.^[^ ^44^ ^]^ In agreement with XRD. ReC/CNT and Re/G displayed a peak at 42.0 and 42.5 eV that could be assigned to Re^+4^ in ReO_2._^[^ ^43^ ^]^

Table 2 shows that the surface atomic ratios of the rhenium samples follow the trend: Re/G > ReOx/G > ReC/G > Re/CNT > ReC/CNT. This indicates that graphite‐supported catalysts exhibit a higher proportion of Re on the surface compared to carbon nanotube‐supported catalysts, in agreement with TEM micrographs. Smaller particle size leads to greater surface coverage by Re species and, consequently, an increased Re/C ratio. This behavior could be attributed to the higher surface area of the graphite over carbon nanotubes, graphitic character, and functional groups.

### 2.6 Catalytic Activity

**Figure 8:**
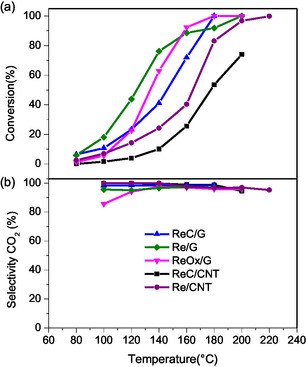
a) Conversion of FA versus temperature and b) CO_2_ selectivity versus temperature.

**Figure** **8a** illustrates the conversion of formic acid (FA) as a function of reaction temperature (80–220 °C) for rhenium‐based catalysts. The figure shows that all the Re catalysts increase the conversion along with the temperature. Among the studied catalysts, those supported on carbon nanotubes consistently exhibited the lowest conversion in all temperatures, with the Re/CNT catalyst being the most active on this support. While pristine support does not show any catalytic activity in the formic acid decomposition, as shown in Figure S11, Supporting Information.

For the graphite‐supported catalysts, the temperature at which a 20% conversion is lower for Re/G, followed by ReC/G and ReOx/G. However, as the temperature increased, the trend changed, and above 160–180 °C, the conversion of ReOx/G and ReC/G eventually surpassed that of the Re/G catalyst. ReC/G and ReOx/G are the first catalysts to achieve 100% conversion. These results suggest that active sites are modified during the reaction.

The selectivity to CO_2_ at each temperature shown in Figure 8b was estimated considering the total concentration of CO_2_ and CO as the only detected products. Overall, the selectivity of CO_2_ formation was higher than MoC phases, as in the literature.^[^ ^18^ ^,^ ^22^ ^]^ The catalysts displayed maximum selectivity in the range of 96–99%. Moreover, it is observed that the lowest selectivity (85% at 100 °C) was observed for ReOx/G catalysts at temperatures below 140 °C, which suggests that its active sites are different from the Re and ReC phases. This behavior may be attributed to the higher concentration of acid sites observed in ReOx/G catalyst through TPD‐NH_3_ analysis. This supports the previously described results, as hydrogen adsorption on oxygen atoms forms additional –OH sites, because the reaction mechanism for the dehydration pathway has been determined to follow the Eley–Rideal model, where a formic acid molecule is dehydrated upon contact with a hydrogen atom adsorbed on the surface of an active site.^[^ ^45^ ^]^ Furthermore, theoretical studies suggest that the most viable mechanism for the dehydrogenation of formic acid on rhenium involves the formation of a formate group (HCOO^−^). This step is exothermic, while the subsequent loss of hydrogen in the second step is endothermic.^[^ ^31^ ^]^ This may explain the increased CO_2_ formation for ReOx as the reaction temperature rises due to the promotion of the endothermic step.

**Figure 9:**
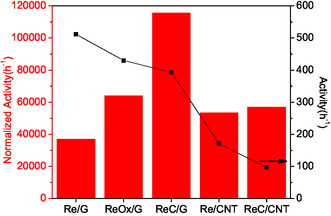
Normalized activity per Re/C atomic surface ratio and activity at 140 °C.

In **Figure** **9**, a comparison between activity at 140 °C and normalized activity per Re/C atomic surface ratio obtained by XPS is shown. The metallic Re phases on both supports exhibit the highest activity for FA conversion at 140 °C. The activity values are consistent with the decrease in mean particle size observed in TEM analyses and the higher Re/C atomic surface ratio determined by XPS. These findings suggest that the dispersion of active sites plays a critical role in the decomposition of formic acid. However, when activity is normalized by the active sites the activity changes, the trend as follows: ReC/G > ReOx/G > ReC/CNT > Re/CNT > Re/G, which suggests that ReC/G catalyst have sites more active than all other catalysts, indicating that rhenium carbide is more active than rhenium in metallic and oxide state. The improved activity of the ReC phase could be attributed to the deformation of the unit cell parameters by the addition of carbon in its structure, as observed in concordance with XRD. A comparison between the literature and the present work is summarized in **Table** **3**.

**Table 3:** Comparison between the present work and the literature on the formic acid decomposition.

Catalyst	Activity at 140 °C (mol CO_2_/mol Metal h^−1^)	Reference
10 Mo_*x*_C/AC	240	[22]
1 Au/SiO_2_	–	[50]
5 Ir/Carbon Norit	–	[47]
1 Pd/Carbon Norit	–	[49]
10 Re/G	512	This work

## 3 Conclusion

Rhenium‐based catalysts supported on carbon in different phases (metallic, carbide, and oxide) were effective for the vapor‐phase dehydrogenation of formic acid, producing CO_2_ and H_2_ as the main reaction products. All catalysts exhibited increased conversion with temperature, with the lowest activity observed for carbon nanotube‐supported catalysts.

Among graphite‐supported catalysts, Re/G initially displayed the highest activity at lower temperatures; however, at temperatures above 160–180 °C, ReOx/G and ReC/G surpassed Re/G, achieving full conversion first. Catalytic performance was strongly influenced by particle size and dispersion. At 140 °C, metallic Re phases exhibited the highest activity, correlating with smaller mean particle sizes (determined by TEM) and higher Re/C atomic surface ratios (determined by XPS). However, the intrinsic activity at lower temperatures followed the trend: ReC/G > ReOx/G > ReC/CNT > Re/CNT > Re/G, suggesting that ReC/G possesses the ReCx phase over the graphite is the most active. ReC/G displayed the highest intrinsic activity, indicating that rhenium carbide is more active than both metallic and oxide Re phases in formic acid decomposition.

Selectivity to CO_2_ was generally high (95–99%), except for ReOx/G, which exhibited lower selectivity (85%) at 100 °C due to its higher acidity, promoting dehydration to CO and H_2_O. As temperature increased, ReOx/G selectivity to CO_2_ improved, likely due to the enhanced endothermic hydrogen loss step. Notably, rhenium catalysts demonstrated higher CO_2_ selectivity than MoC‐based catalysts reported in the literature.

These findings highlight the importance of Re species treatment in achieving optimal catalytic performance and the relevance of rhenium in the dehydrogenation of formic acid.

## 4 Experimental Section

4.1

4.1.1

Commercial high surface area graphite (denoted as G; Timrex 400, purity >95%), and O‐functionalized multiwalled carbon nanotubes (denoted as CNT; cheaptubes, 99% purity, and 7% functional content).

Catalysts were prepared by incipient wetness impregnation followed by different treatments. Briefly, the amount of precursor salt (NH_4_ReO_4_, Molymet S.A.) required to achieve a nominal 10% Re content was dissolved in water. The resulting aqueous solution was impregnated onto the carbon supports, either high‐surface‐area graphite (G) or carbon nanotubes (CNT). The mixture was aged for 6 h at room temperature and subsequently dried at 100 °C overnight. For the Re‐impregnated CNT samples, the material was divided into two parts: the first part was carburized in a 25/75% V/V flow of C_2_H_4_/H_2_ for 90 min at 650 °C (5 °C min^−1^) and labeled as ReC/CNT. The second part was reduced to a 100% H_2_ flow for 90 min at 650 °C (5 °C min^−1^) and labeled as Re/CNT. For the Re‐impregnated G samples, the material was aged for 6 h at room temperature and dried at 100 °C an then divided into three parts: one part was carburized under a 25/75% V/V flow of C_2_H_4_/H_2_ for 90 min at 650 °C (5 °C min^−1^) and labeled as ReC/G. The second part was reduced under a H_2_ atmosphere for 90 min at 650 °C (5 °C min^−1^) and labeled as Re/G. Finally, the third part was treated under N_2_ atmosphere for 90 min at 350 °C and labeled as ReOx/G. All gases were used with a total flow of 100 mL min^−1^.

##### Characterization

The textural properties of the catalysts were measured from the nitrogen adsorption–desorption isotherms at −196 °C using a Micromeritics 3Flex instrument. Before analysis, 10 mg of each sample was degassed for 4 h at 110 °C under vacuum using a Micromeritics SmartVacPrep system. Surface area calculations were based on the Brunauer–Emmett–Teller (BET) theory and followed the Rouquerol criteria.^[^ ^46^ ^]^ The total pore volume was determined as the single‐point pore volume at *p*/*p*_0_ = 0.99, and pore size distribution was determined with the BJH method using the desorption branch. The reducibility of the in situ treated samples by reduction (Re/G, Re/CNT), carburization (ReC/G, ReC/CNT) and calcination (ReOx/G) materials were evaluated using hydrogen temperature‐programmed reduction (H_2_‐TPR) on a Micromeritics 3Flex instrument coupled with a Cirrus 2 mass spectrometer (MKS Spectra Product) and a thermal conductivity detector (TCD). Samples weighing between 15 and 20 mg were loaded into a quartz reactor tube and heated from room temperature to 1000 °C at a rate of 10 °C min^−^^1^ under a 5% H_2_/Ar flow (100 mL min^−1^). The gas stream was passed through a cold trap immersed in a mixture of isopropanol and liquid nitrogen before entering the mass spectrometer and TCD. Mass signals corresponding to CO (*m*/*z* = 28), CO_2_ (*m*/*z* = 44), and CH_4_ (*m*/*z* = 16) were monitored.

Temperature‐programmed reaction with methanol (TPRe‐MeOH) was carried out using the same Micromeritics 3Flex instrument coupled to a mass spectrometer. For this analysis, 50 mg of sample was pretreated under helium flow (50 mL min^−1^) at 300 °C with a heating rate of 10 °C min^−1^ for 1 h. Methanol adsorption was then conducted by saturating the catalyst with a helium/methanol mixture at room temperature for 30 min. The solid was purged with helium until the TCD baseline stabilized before starting the temperature‐programmed reaction. The products formed during the reaction were analyzed by mass spectrometry, with dimethyl ether (DME) indicating acidic sites, CO and CO_2_ indicating basic sites, and formaldehyde (HCOH) indicating redox sites. All TPRe‐MeOH MS signals were standardized by catalyst mass and on the same scale. For the temperature‐programmed desorption of NH_3_ (TPD‐NH_3_), the sample underwent initial pretreatment for 30 min at 350 °C (10 °C min^−1^) under a flow of He (50 mL min^−1^). Subsequently, the adsorption of NH_3_ was conducted at 100 °C for 15 min (30 mL min^−1^), followed by desorption under He (100 mL min^−1^) at a rate of 10 °C min^−1^ until reaching 500 °C. Total acid sites were quantified by area integration. Transmission electron microscopy (TEM) images of the rhenium‐based catalysts were obtained using a Jeol JEM‐1200 EXII system. Samples were ground and dispersed in methanol and then transferred to a copper grid using the methanol dispersion method. The particle size distribution of rhenium was determined by analyzing at least 10 different TEM micrographs per catalyst. More than 200 particles were measured using Image Tool 3.0 software to construct histograms. The average particle diameter *d*_p_ was calculated using the following Equation (1)

(1) $$d_{p}=\sum_{i}^{n}\frac{d_{i}}{n}$$

X‐ray diffraction (PXRD) patterns were recorded using a Bruker D2 Phaser diffractometer with Cu Kα radiation (*λ *= 1.54 Å). Identification of the phases in the powder diffraction patterns was achieved using EVA diffraction file data. The X‐ray source was operated at 30 kV and 10 mA. XPS was performed to analyze the chemical states of the samples at the surface. Measurements were conducted using a Perkin Elmer PHI 1257 XPS‐Auger spectrometer operating in an ultra‐high vacuum chamber with a hemispherical electron energy analyzer. The X‐ray source, consisting of an Al anode (*h* *ν* = 1486.6 eV), provided unfiltered Kα radiation. The main spectrometer chamber pressure was maintained at ≈10^−7^ Pa during data acquisition. The binding energy (BE) scale was calibrated using the C 1s peak of the carbon support, set to 284.8 eV.

##### Catalytic Experiments

The thermal decomposition reactions of formic acid (FA) were conducted in the gas phase using a fixed‐bed reactor under continuous flow conditions. A 6% FA/He mixture was fed into the reactor at a total flow rate of 25 cm^3^ min^−1^ through a thermostatically controlled saturator maintained at 15 °C. The concentration of formic acid in the helium flow was calculated using the Antoine equation, with the Antoine Parameters A, B, and C obtained from the NIST Database.

The catalysts were loaded into a quartz U‐shaped reactor, packed between two layers of silicon carbide, and secured with quartz wool. To minimize internal diffusion limitations, the catalysts were sieved to a particle size range of 350–500 µm. The reaction products were analyzed using gas chromatography (GC).

The concentrations of CO and CO_2_ were calculated by (Equation 2)

(2) $$\left[\mathrm{CO}\right]=\frac{\text{area}\left[\mathrm{CO}\right]}{\mathrm{RF}};\left[{\mathrm{CO}}_{2}\right]=\frac{\text{area}\left[{\mathrm{CO}}_{2}\right]}{\mathrm{RF}}$$

where RF is the response factor. Since no other products were observed, the total conversion of formic acid was determined as the sum of CO and CO_2_ concentrations related to the initial concentration of formic acid. ${\left[\text{HCOOH}\right]}_{0}\;=\;{\left[\mathrm{CO}\right]}_{m}\;+\;{\left[{\mathrm{CO}}_{2}\right]}_{m}$ at *T*_max_ to obtain a complete conversion of HCOOH. HCOOH consumption was followed to ensure the effective conversion to the products.

To determine the conversion of formic acid, the concentrations of CO and CO_2_ were calculated using the following equation. It was assumed that CO_2_ was present in a CO_2_/H_2_ ratio of 1 as has been informed in literature.^[^ ^47^ ^]^ This assumption was based on the fact that He, used as the reference gas in the TCD analysis, has a thermal conductivity similar to H_2_, as the concentration of H_2_ obtained is inside the detection limit; it was not observed.

(3) $$X_{\text{HCOOH}}=\frac{\left[\mathrm{CO}\right]+\left[{\mathrm{CO}}_{2}\right]}{{\left[\text{HCOOH}\right]}_{0}}\times 100$$

where $X_{\text{HCOOH}}$ is the conversion of formic acid.

The selectivity of CO_2_ and CO were calculated by the (Equation 4) based on Tao et al.^[^ ^48^ ^]^ and Ross et al.^[^ ^49^ ^]^ since CO_2_ is related to H_2_ formation, while CO selectivity is related to H_2_O

(4) $${\mathrm{CO}}_{2}\;\text{Selectivity}=\frac{\left[{\mathrm{CO}}_{2}\right]}{\left[\mathrm{CO}\right]+\left[{\mathrm{CO}}_{2}\right]}\times 100$$

According to Carrales et al.^[^ ^22^ ^]^ Activity at 140 °C was calculated from (Equation 5)

(5) ACO2=[CO2]molRe

where mol Re = rhenium moles in the mass of the catalyst charged.

## Conflict of Interest

The authors declare no conflict of interest.

## Supporting information

Supplementary Material

## Data Availability

The data that support the findings of this study are available from the corresponding author upon reasonable request.
