# Supplementary material for: Selective Vapor‐Phase Formic Acid Decomposition Over Carbon‐Supported Rhenium Catalysts with Metallic, Carbide, and Oxide Rhenium Phases
Source: ChemistryOpen. 2025 Sep 22;14(12):e202500412. doi: 10.1002/open.202500412 (PMC12680561; doi:10.1002/open.202500412)
Supplement: Supplementary file 1 — Supplementary Material [file OPEN-14-e202500412-s001.pdf]

Supporting Information

Claudio Contreras-Díaz [a,b], Verónica Naharro-Ovejero [c], Claudio Araya-López [a,b], Juan Seguel [a,b], Marcos Flores [d], Vicente Diaz [d], Néstor Escalona *[a,b,e], AB Dongil *[c]

[a] Claudio Contreras-Díaz, Dr. Claudio Araya-López, Dr. Juan Seguel, Prof. Dr. Néstor Escalona*

Departamento de Ingeniería Química y Bioprocesos

Pontificia Universidad Católica de Chile

Avenida Vicuña Mackenna 4860, Macul, Santiago, Chile Chile

E-mail: neescalona@uc.cl

[b] Claudio Contreras-Díaz, Dr. Claudio Araya-López, Dr. Juan Seguel, Prof. Dr. Néstor Escalona*

Millennium Nuclei on Catalytic Processes Towards Sustainable Chemistry (CSC) Chile

[c] Verónica Naharro-Ovejero

Instituto de Catálisis y Petroleoquímica, CSIC

Cantoblanco, 28049 Madrid, Spain

[d] Dr. Marcos Flores, Vicente Diaz

Departamento de Física. Facultad de Ciencias Físicas y Matemáticas, Universidad de Chile

Avenida Blanco Encalada 2008, Santiago, Chile

[e] Prof. Dr. Néstor Escalona*

Departamento de Química Física

Facultad de Química y de Farmacia, Pontificia Universidad Católica de Chile

Santiago 7820436, Chile

[c] Dr. AB Dongil*

Instituto de Catálisis y Petroleoquímica, CSIC

Cantoblanco, 28049 Madrid, Spain

E-mail: a.dongil@csic.es

Figure S1.a shows an intense peak at the beginning, followed by a gradual decrease. This initial peak is attributed to an artifact of the technique, which is formed at the moment of

closing the hysteresis loop and cannot be considered as a pore of the material. **Fig S1.b** shows a decrease in dV/dlogw from pristine support as well.

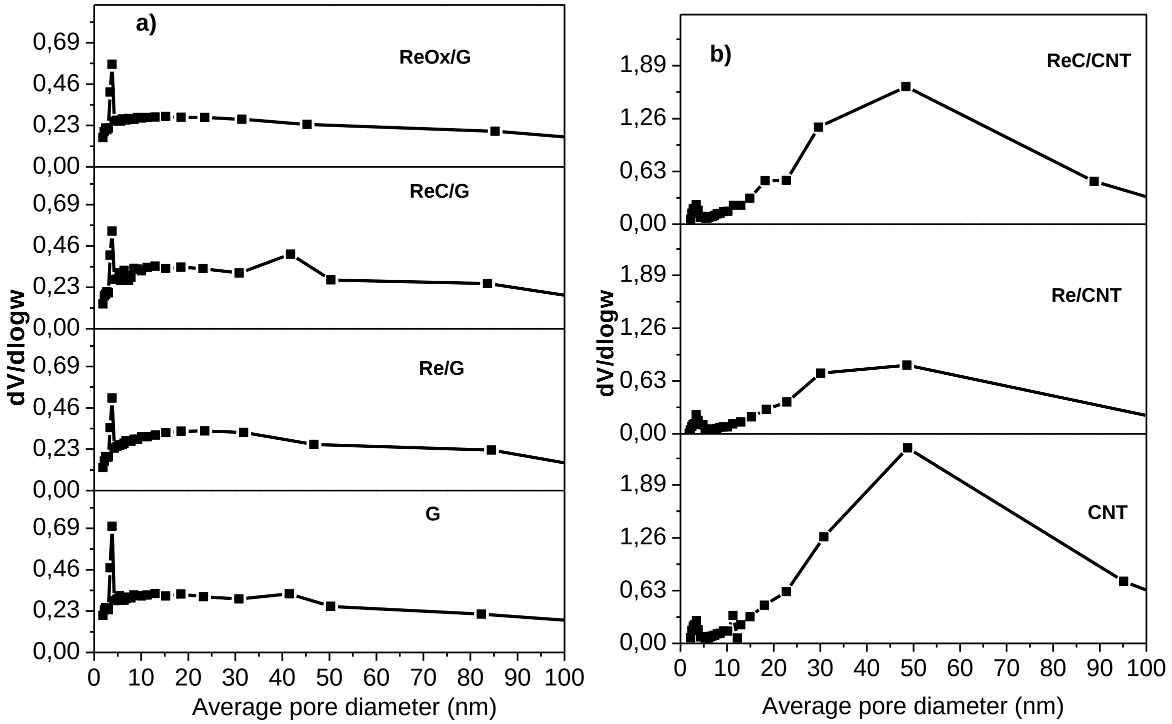

Figure S1. Pore size distribution for Re-based catalysts supported on a) graphite and b) carbon nanotubes.

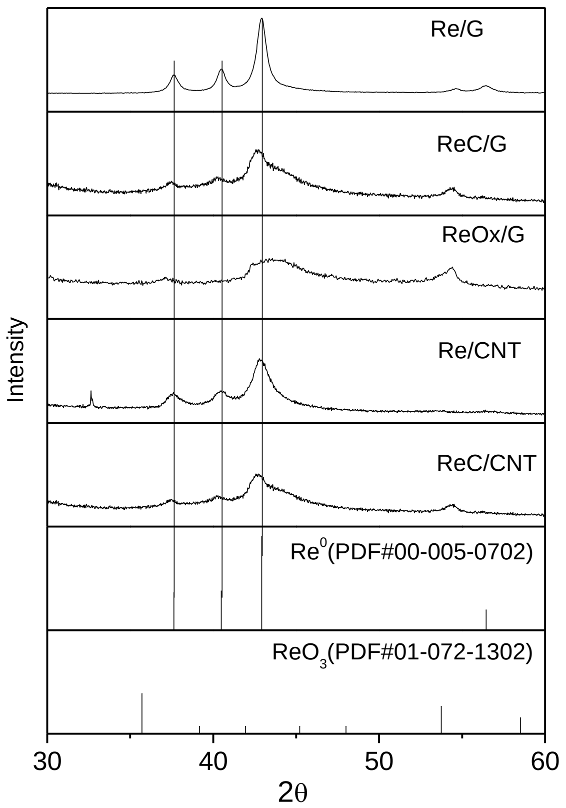

Figure S2. amplied diffraction pattern of the catalysts

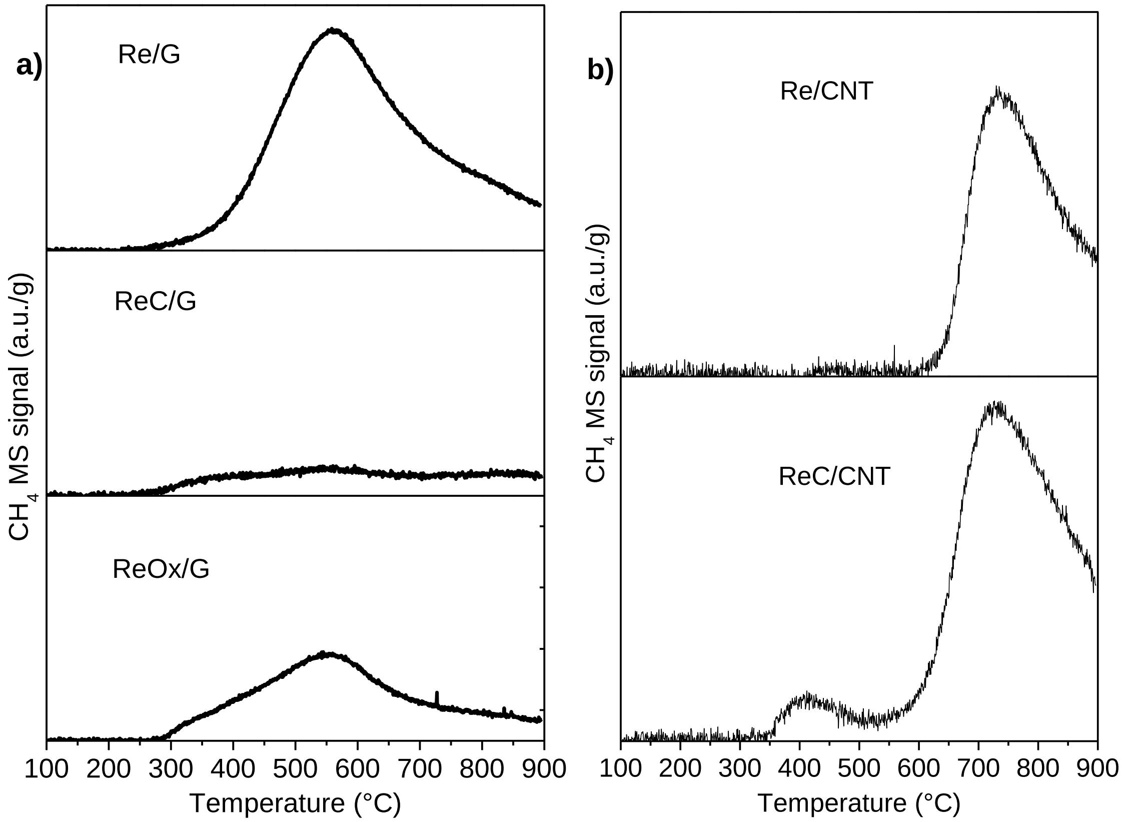

Figure S3. CH_4-MS signal of rhenium-based catalysts supported on a) graphite and b) carbon nanotubes.

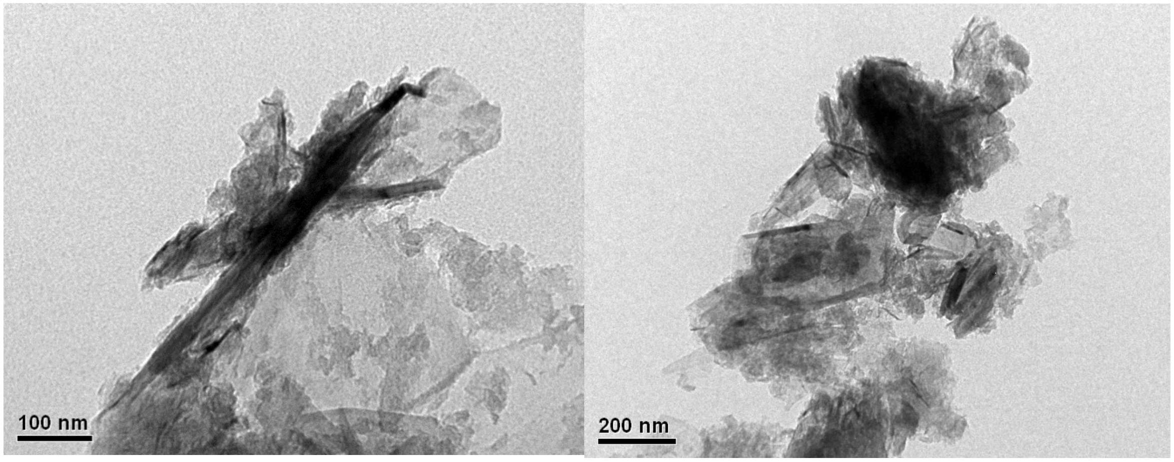

Figure S4. TEM images of ReOx/G catalyst

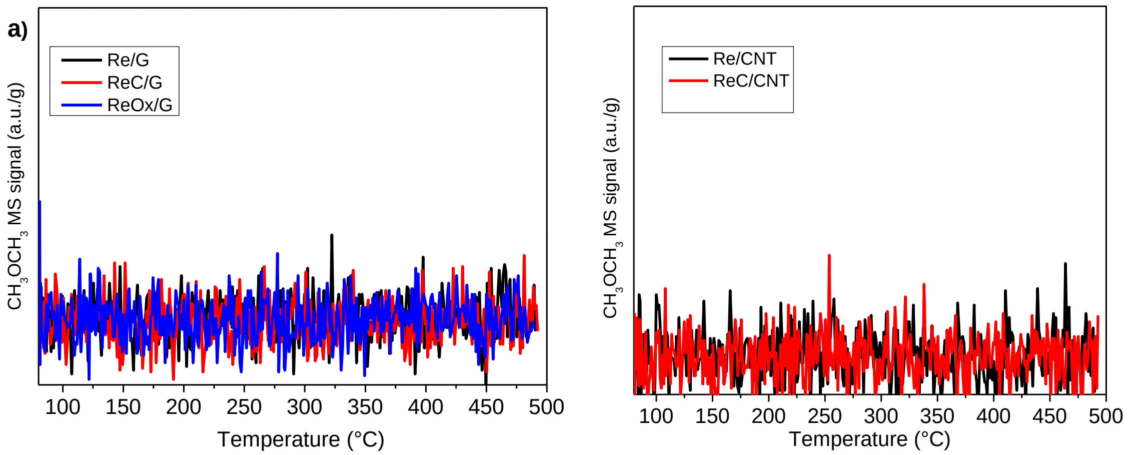

Figure S5. 46 m/z MS signal of CH_3OCH_3 from TPRe-MetOH of Re-based catalysts supported on a) graphite and b) carbon nanotubes.

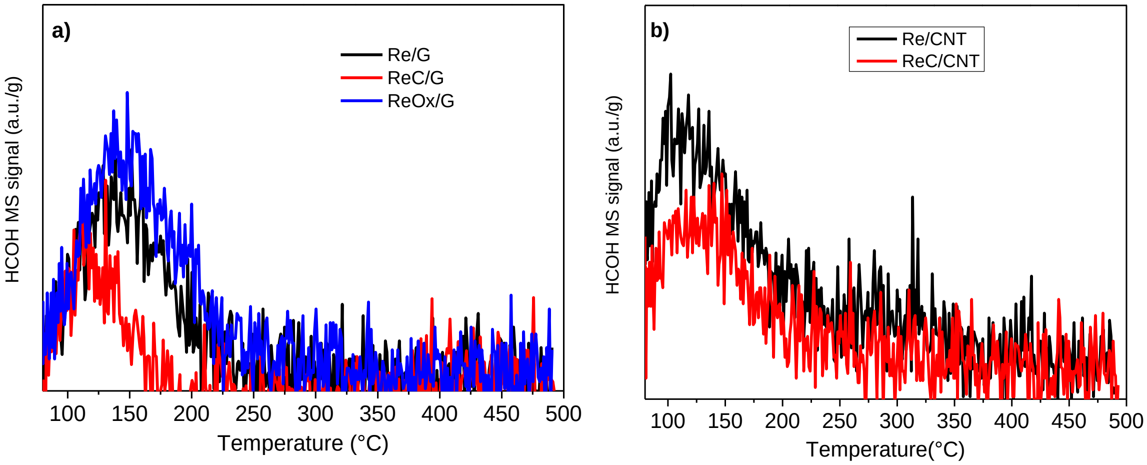

Figure S6. 30 m/z MS signal of HCOH from TPRe-MetOH of Re-based catalysts supported on a) G and b) CNT.

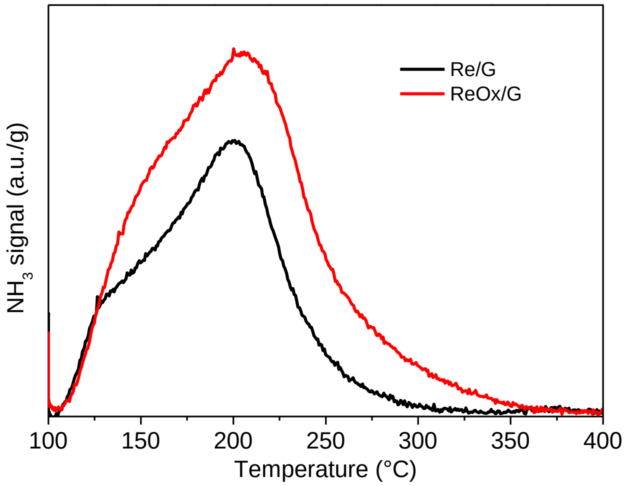

Figure S7. Temperature-programmed desorption-NH_3 of rhenium-based catalysts.

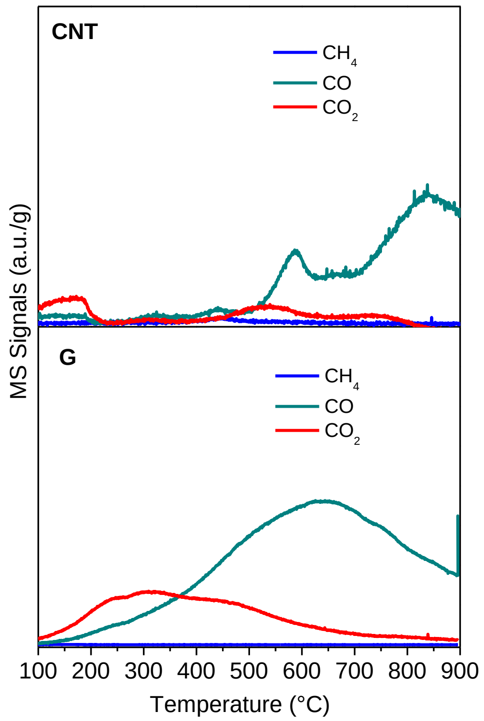

Figure S8. TPD-He of the pristine supports

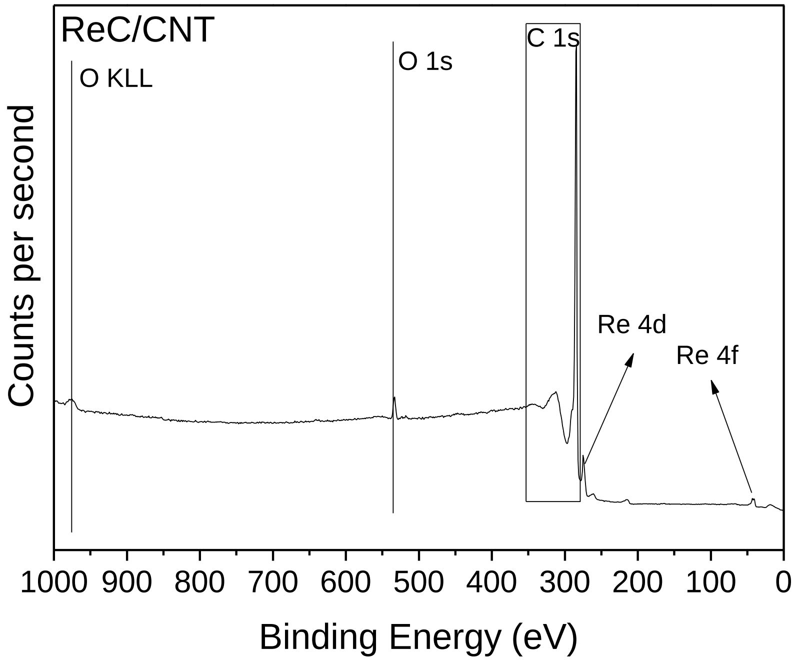

Figure S9. XPS Survey of ReC/CNT

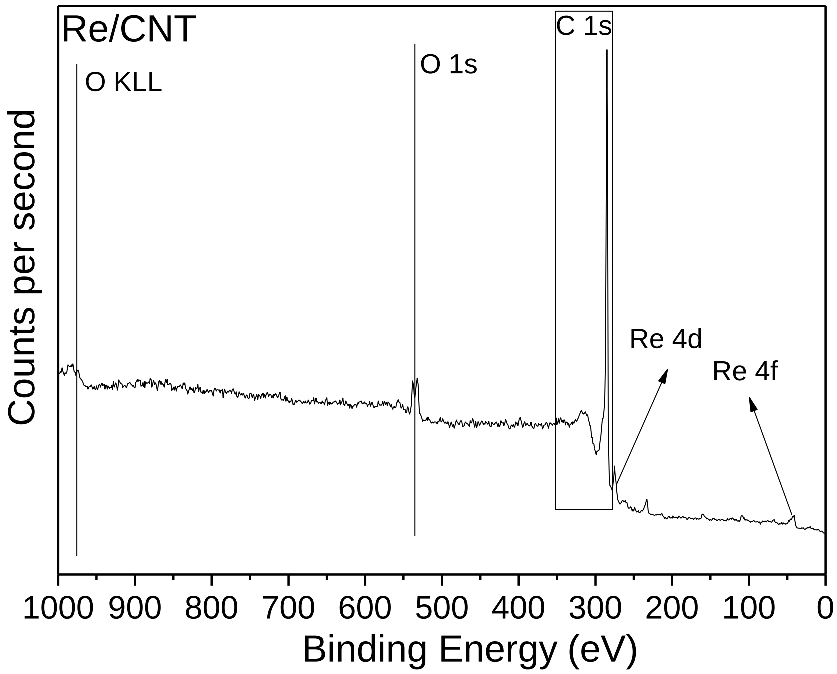

Figure S10. XPS Survey of Re/CNT

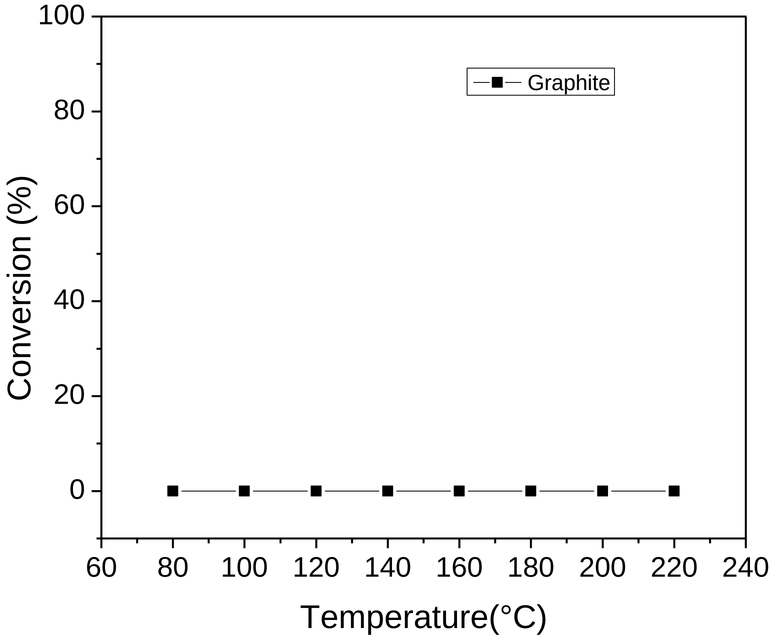

Figure S11. Conversion of formic acid over graphite

Table S1. Total acid sites of Re/G and ReOx/G catalysts.

Samples	Total Acid Sites (10^{-5} mol $NH_3 \cdot g^{-1}$)
Re/G	1.1
ReOx/G	1.9
